# Invasive Lobular Carcinoma of the Male Breast With BRCA2 Mutation

**DOI:** 10.1155/crip/3580444

**Published:** 2026-02-26

**Authors:** Misaki Abe, Fumi Saito, Naoko Honma, Yuko Tamaki, Mayu Goto, Katsunori Fukutake, Satoshi Sonobe, Yukiko Katagiri, Tomoko Shibayama, Hideaki Ogata

**Affiliations:** ^1^ Division of Breast and Endocrine Surgery, Department of Surgery, Toho University School of Medicine, Ota-ku, Tokyo, Japan, toho-u.ac.jp; ^2^ Department of Surgical Pathology, Toho University School of Medicine, Ota-ku, Tokyo, Japan, toho-u.ac.jp; ^3^ Division of Clinical Genetics, Toho University School of Medicine, Ota-ku, Tokyo, Japan, toho-u.ac.jp; ^4^ Department of Orthopedic Surgery, Toho University School of Medicine, Ota-ku, Tokyo, Japan, toho-u.ac.jp

**Keywords:** bilateral synchronous, BRCA2 mutation, invasive lobular carcinoma, male breast cancer

## Abstract

Male breast cancer (MBC) is a rare condition, accounting for < 1% of all breast cancer cases. Reports of bilateral synchronous MBC are even more uncommon. Although lobular carcinoma is generally absent in the male mammary gland, a few cases of lobular carcinoma in MBC have been documented, comprising 1%–2% of all MBC cases. A man in his 80s presented to our hospital with a mass on his left nipple. After detailed examination, he was diagnosed with invasive ductal carcinoma of the left breast and invasive lobular carcinoma of the right breast. Because he had a family history of breast cancer, he underwent genetic testing and was found to have a *BRCA2* gene mutation (c.331_347delinsC [p.Asn111Leufs∗5]). Simultaneous surgery was performed for bilateral breast cancer. Although drug therapy and radiation therapy were recommended after the operation, the patient was under observation due to his advanced age. A brief literature review is presented in this section.

## 1. Background

Male breast cancer (MBC) is a rare condition and accounts for < 1% of all breast cancers. Although lobular structures are infrequent in the male breast, rare cases of invasive lobular carcinoma (ILC) have been described representing only 1%–2% of cases [1].

Contralateral breast cancer diagnosed within 12 months of a prior breast cancer diagnosis is known as bilateral synchronous breast cancer [2]. Bilateral synchronous breast cancer is also extremely rare, constituting 0.5%–2.5% of MBC [3, 4].

Moreover, in recent years, the link between MBC and *BRCA2* gene mutations has become clear.

To the best of our knowledge, only a few studies [5] have described a bilateral synchronous breast cancer with ILC for MBC.

This report is aimed at raising awareness about this rare entity by presenting the clinical and pathological features of a man in his 80s with bilateral synchronous breast cancer and ILC.

## 2. Case Presentation

A man in his 80s was admitted to our hospital complaining of bleeding and a painless, palpable tumor in his left breast. He had a clinical history of giant cell tumor (GCT) of the fifth lumbar vertebra. He was administered denosumab for a total of 47 times over 4 years as treatment for GCT. As a result of the treatment, he developed jaw osteonecrosis. The patient had no history of radiation therapy or liver dysfunction. However, the patient had a family history of malignancy: two of his sisters and one niece had breast cancer, and another sister had uterine cancer.

**Figure 1:**
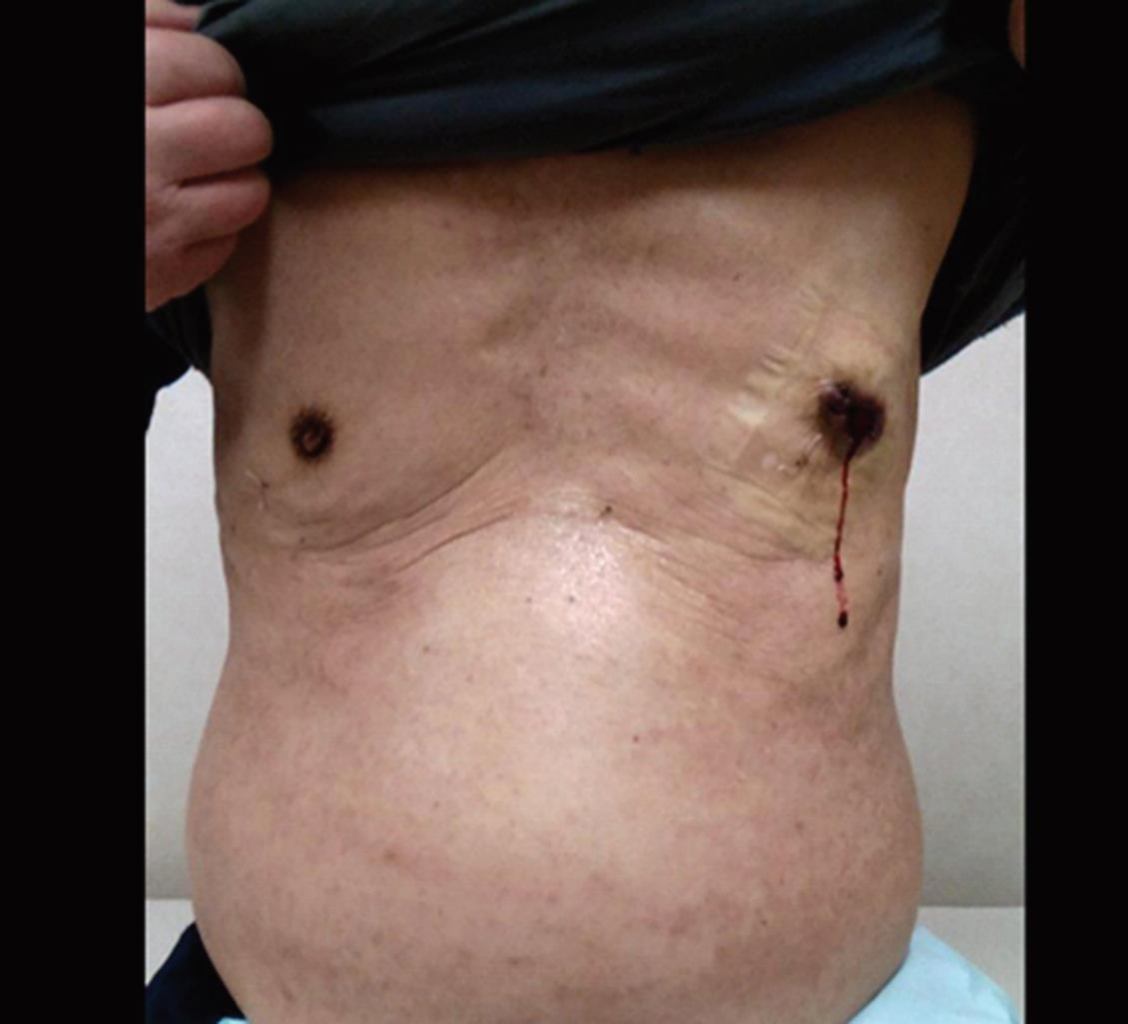
Examination findings; ulceration with bleeding in the left chest.

Physical examination revealed a poorly mobile tumor with bleeding and an ulcer on his left chest (Figure 1). The mass in the right breast was not palpable.

**Figure 2:**
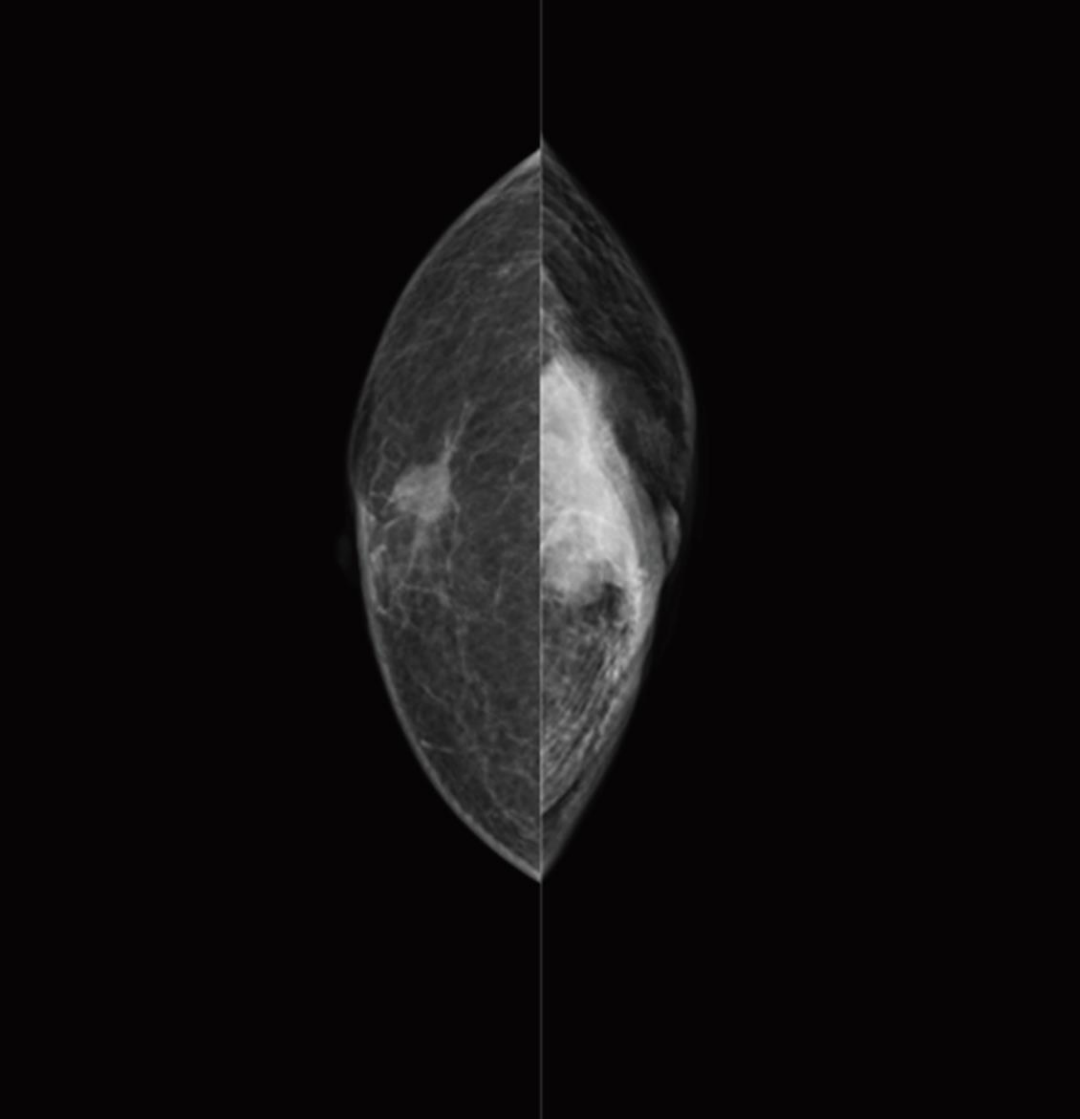
Mammography findings (CC). Lt: High‐density irregular mass with skin retraction and infiltration of the pectoralis major muscle. Rt: Isodose mass near the nipple.

The Left craniocaudal (CC) mammographic (MMG) projections showed a high‐density irregular mass occupying the entire breast, with skin retraction and infiltration of the pectoralis major muscle on the left breast. On the right MMG, isodose mass was identified near the nipple (Figure 2).

Breast ultrasonography revealed an irregular, solid mass measuring 38 × 27 mm, which was suspected to be infiltrating the pectoralis major muscle and skin centered on the left nipple (Figure 3a). In the right breast, an irregular margin mass of 15 × 10 mm was observed near the nipple (Figure 3b). Bilateral axial lymph nodes showed no suspicious cancer metastases. A full‐body examination was performed, and no findings indicating distant metastases were observed.

Figure 3Breast ultrasound findings.

**Figure:**
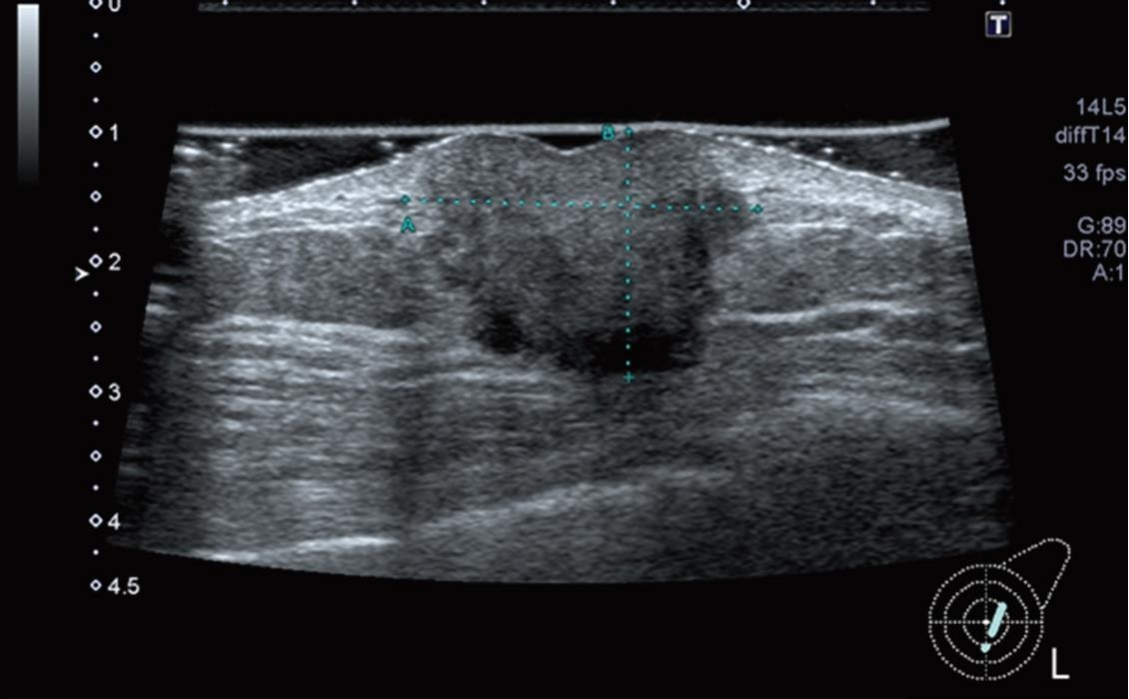
(a) Lt: 38 × 27 mm suspected skin and pectoralis major muscle invasion

**Figure:**
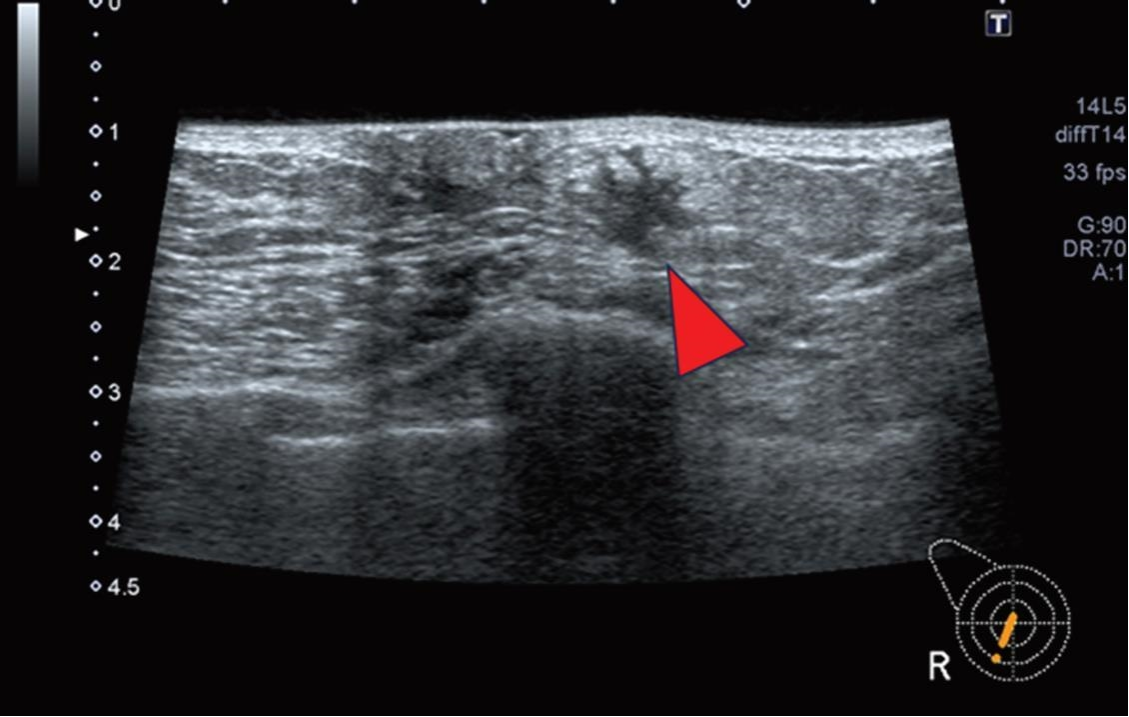
(b) Rt: 15 × 10 mm irregular mass near the nipple (arrowhead)

Ultrasound‐guided core needle biopsy revealed an invasive ductal carcinoma (IDC) on the left side (cT4N0M0, clinical stage IIB, ER/PR positive, HER2 negative, histological Grade 1, and E‐cadherin positive) and an ILC on the right side (cT1N0M0, clinical stage I, ER/PR positive, HER2 negative, and E‐cadherin negative).

Owing to a strong family history of breast cancer, preoperative genetic testing was recommended. As the result of the gene test, he had a *BRCA2* likely pathogenic variant (c.331_347delinsC [p.Asn111Leufs∗5]). This frameshift mutation is predicted to result in premature truncation of the *BRCA2* protein at amino acid Position 115.

The patient underwent a bilateral breast mastectomy with sentinel node biopsy. The surgery was performed on both sides simultaneously. Two sentinel lymph nodes were removed from the right side, and intraoperative rapid diagnosis revealed no cancer metastasis. On the other hand, three sentinel lymph nodes were removed from the left side, and cancer metastasis was found in one of them. As a result, we performed an additional left axillary lymph node dissection. Six lymph nodes were removed during the left axillary lymph node dissection.

**Figure 4:**
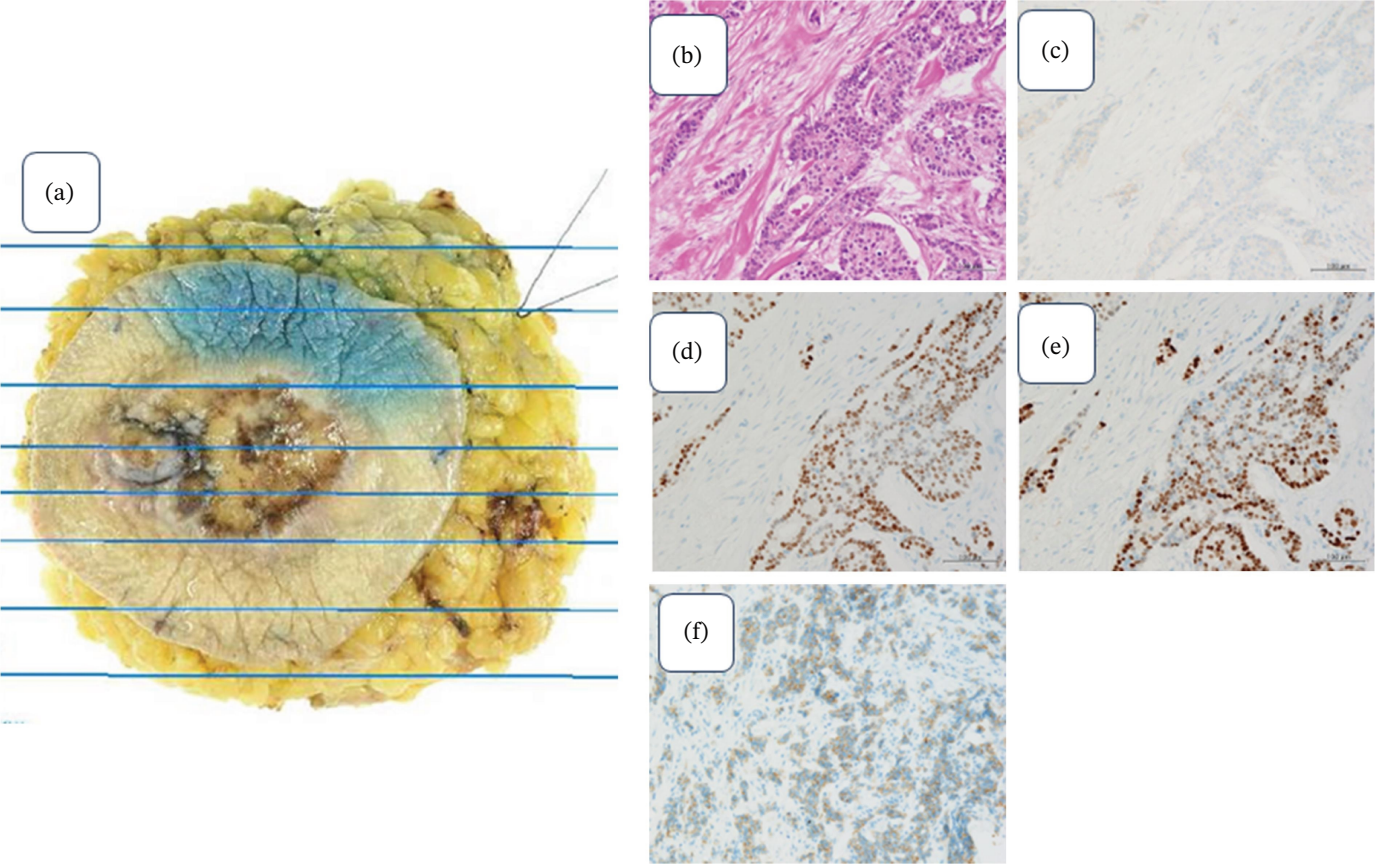
Lt breast surgical specimen. (a) Tumor size: 39 × 18 mm ulcer formation is observed around the nipple. (b) H and E stained ×400: Atypical cell proli ferate in a solid tumor. (c) HER2 (×400): 1+. (d) ER (×400): PS5 + IS2 = TS7. (e) PgR (×400): PS5 + IS2 = TS7. (f) E‐cadherin (×400): Positive.

**Figure 5:**
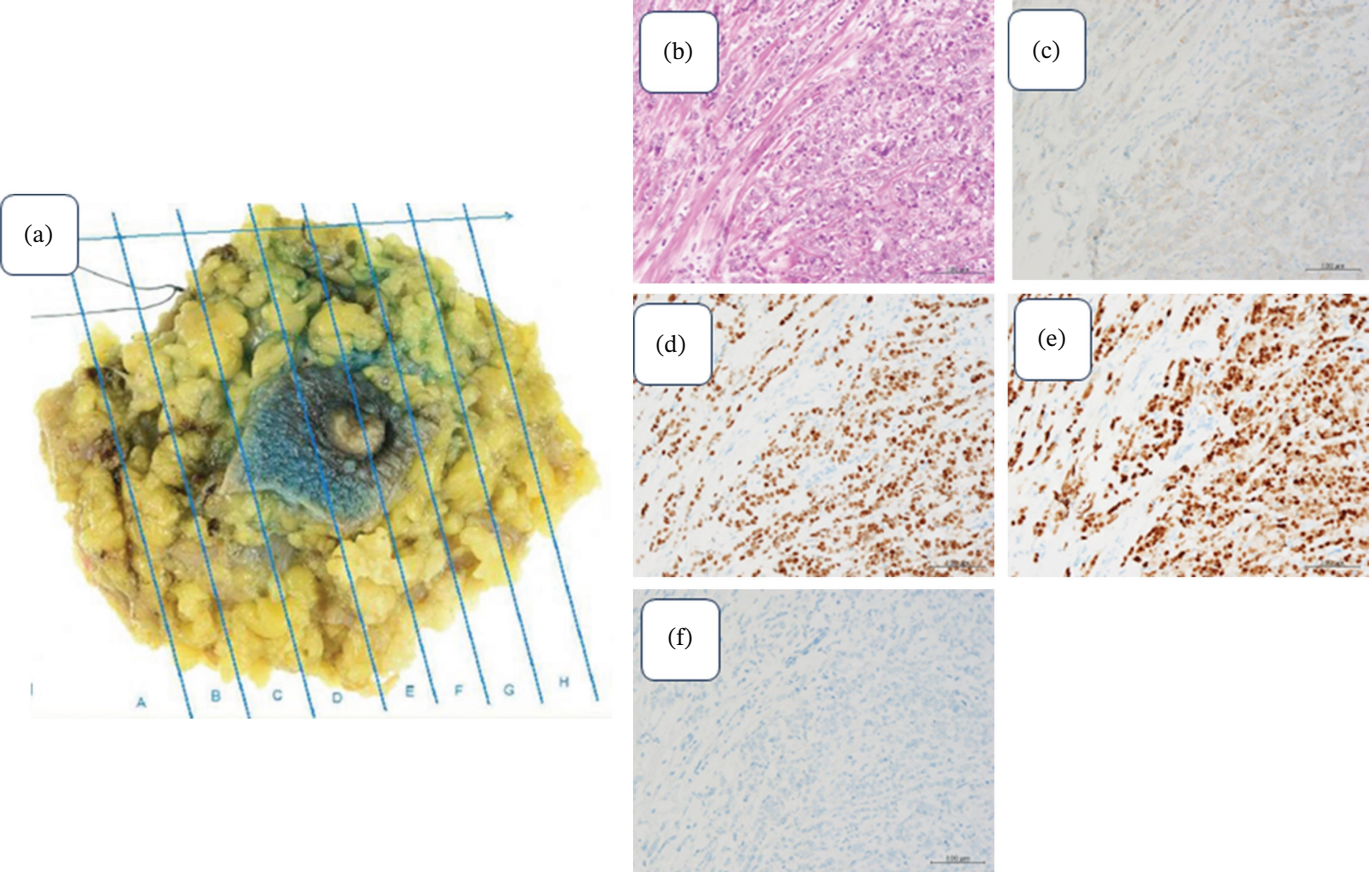
Rt breast surgical specimen. (a) Tumor size: 14 × 12 mm tumor was found near the nipple. (b) H and E stained (×400): Cell with high N/C ratio proliferation in a cord‐like fashion. (c) HER2 (×400): 1+. (d)ER (×400): PS5 + IS2 = TS7. (e) PgR (×400): PS5 + IS2 = TS7. (f) E‐cadherin (×400): Negative.

Ultimately, metastasis was observed in one of the left axillary lymph nodes, and its size was 5 mm. Pathological examination of the surgical materials confirmed IDC on the left side (pT4, N1a, pStage IIIB, histological Grade III, ly1,v1, ER/PR‐positive, HER2 negative, Ki‐67 40%, and negative surgical margin) (Figures 4a, 4b, 4c, 4d, 4e, and 4f) and ILC on the right side (pT1c, pN0, pStageI, ly1,v1 ER/PR‐positive, HER2 negative, Ki‐67 25%, solid type, and negative surgical margin) (Figures 5a, 5b, 5c, 5d, 5e, and 5f).

Because of the *BRCA2* mutation, counseling was provided to the patient and their families, and the patient was screened for prostate and pancreatic cancers. His prostate specific antigen (PSA) level was 3.370 ng/mL, and at this stage, prostate cancer had not developed. However, it was decided that his PSA levels will continue to be measured regularly in the future. One of his two children was found to have a *BRCA* gene mutation. His sister, who has already developed breast cancer, is elderly and has not undergone testing, but she is highly likely to have a *BRCA* gene mutation.

## 3. Discussion

Although MBC is rare, accounting for < 1.0% of all breast cancers, its incidence has been increasing in Japan, Europe, and the United States [1]. Similar to other cancers, advanced age is a risk factor for MBC. Hormonal imbalances, particularly elevated levels of female hormones, have also been implicated in its development. These include obesity, Klinefelter syndrome, drugs, and exogenous hormones (e.g., gender reassignments) [6]. The average age at diagnosis is approximately 70 years in men and 60 years in women. Furthermore, MBC is often diagnosed at an advanced stage [7]. Many male patients do not initially believe they are at risk of developing breast cancer.

MBC is frequently reported to be hormone receptor (HR)‐positive, and strongly positive cases reportedly have long overall and recurrence‐free survival. Yousef et al. reported that age, hormonal imbalance, *BRCA* mutation, race, radiation exposure, and family history of breast cancer are the most significant risk factors for the development of MBC [8]. In this case, family history, age, and *BRCA2* mutations were risk factors for MBC. As 10% of MBC cases have a *BRCA2* genetic mutation [9], the American Society of Clinical Oncology (ASCO) recommends counseling for all MBC [10]. It is known that prostate cancer that develops in men with *BRCA2* mutations tends to be highly aggressive. Therefore, surveillance through measuring PSA level is recommended for them.

Although MBC is rare, bilateral MBC is rare and accounts for 0.5%–2.5% of all MBC cases [3, 4]. Lobular carcinoma of the female breast is relatively common, accounting for approximately 15% of all cases of female breast cancer. This pathological type is extremely rare in male because lobules and acini are not found in normal male breast [11].

In the normal female breast, tissue is consists of acini or lobules and ducts. The inner luminal layer of epithelial cells lining the acini is round and regular. They surround a central open space creating a gland‐like appearance. Surrounding the epithelial lining are myoepithelial cells that form the basement membrane. It is the epithelial cells that give rise to lobular carcinoma. These malignant cells fill the acini and invade surrounding tissue in the classic Indian single‐life appearance. Basil et al. have reported that once male breasts are exposed to estrogen, true acini and lobules are formed, and they can change to resemble female breasts [11].

Evaluation of 2537 MBC cases from the Surveillance, Epidemiology, and End Results (SEER) program database between 1973 and 1998 revealed a 1.5% ILC [12]. Pathogenic *BRCA* alterations were detected in approximately 16% of all MBC cases, with 12.5% found in *BRCA2* [13]. Silvestri et al. [14] analyzed 326 MBC with *BRCA2* pathogenic mutations and found that lobular carcinoma accounted for only 1.5% of the cases.

Generally, IDC is associated with mutations in *BRCA1* and *TP53*; *BRCA2* mutations are common in both IDC and ILC, and the *CDH1* gene is more common in ILC. Although ILC is usually of low historical grade, with a low Ki‐67/mitotic index and HR positivity, patient outcomes have been reported to be poorer than those in men with IDC, given the invasive nature and tendency for widespread metastatic disease [15].

Prostate cancer that occurs in patients with *BRCA2* gene mutations is known to be highly aggressive, and surveillance is necessary [16]. On the other hand, the relative risk of developing pancreatic cancer in *BRCA2* carrier patients has been reported to be three to four times higher. Therefore, surveillance is recommended only for those who have a first‐degree relative with pancreatic cancer [17]. As a result, it was not conducted in this case.

The implementation rate of counseling for relatives of individuals with hereditary tumors is reported to be 48% (95% CI: 38–58), and cascade testing is reported to be 41% (95% CI: 34–48) [18]. This data is believed to be greatly influenced by cultural and social backgrounds.

In conclusion, we reported a case of ILC in a male breast with a *BRCA2* mutation. Although ILC is extremely rare in MBC, it should be considered. In the case of MBC, the onset is likely due to genetic factors, and genetic testing should be recommended following ASCO guidelines. If a genetic mutation is detected, not only is screening for related diseases required for the patient, but testing should also be considered for their relatives.

## Author Contributions

Fumi Saito: organized the study conception; Misaki Abe: wrote the manuscript; Naoko Honma and Satoshi Sonobe: summarized pathological findings; Yuko Tamaki and Yukiko Katagiri: summarized genetic findings; Mayu Goto, Katsunori Fukutake, and Tomoko Shibayama: revision of manuscript; Hideaki Ogata: final approval of manuscript.

## Funding

No funding was received for this manuscript.

## Ethics Statement

This study was conducted in accordance with the Declaration of Helsinki and was approved by the Institutional Review Board of Toho University.

## Consent

Consent was obtained directly from the patient.

## Conflicts of Interest

The authors declare no conflicts of interest.

## Data Availability

The datasets are available from the corresponding author on reasonable request.
